# Dispelling myths about rare disease registry system development

**DOI:** 10.1186/1751-0473-8-21

**Published:** 2013-10-16

**Authors:** Matthew Bellgard, Christophe Beroud, Kay Parkinson, Tess Harris, Segolene Ayme, Gareth Baynam, Tarun Weeramanthri, Hugh Dawkins, Adam Hunter

**Affiliations:** 1 Centre for Comparative Genomics, Murdoch University, Perth 6150, Western Australia; 2 Aix-Marseille Université, Faculté de Médecine de la Timone, INSERM UMR 910, Marseille, France; 3 AP-HM, Département de Génétique Médicale, Hôpital d’enfants Timone, Marseille, France; 4 Alstrom Syndrome, 49 Southfield Ave, Paignton S, Devon, TQ3 1LH, UK; 5 Polycystic Kidney Disease Charity (UK), PKD International, Ciliopathy Alliance, London, UK; 6 INSERM US14, ORPHANET 96 rue Didot, Paris 75014, France; 7 Genetic Services of Western Australia, King Edward Memorial Hospital, Perth, Western Australia; 8 School of Paediatrics and Child Health, University of Western Australia, Perth, Western Australia; 9 Institute for Immunology and Infectious Diseases, Murdoch University, Perth, Western Australia; 10 Office of Population Health Genomics, Public Health and Clinical Services Division, Department of Health, Government of Western Australia, Perth, Western Australia; 11 School of Pathology & Laboratory Medicine, University of Western Australia, Perth, Western Australia; 12 Curtin Health Innovation Research Institute, Curtin University of Technology, Perth, Western Australia

**Keywords:** Rare disease, Disease registry, Software development

## Abstract

Rare disease registries (RDRs) are an essential tool to improve knowledge and monitor interventions for rare diseases. If designed appropriately, patient and disease related information captured within them can become the cornerstone for effective diagnosis and new therapies. Surprisingly however, registries possess a diverse range of functionality, operate in different, often-times incompatible, software environments and serve various, and sometimes incongruous, purposes. Given the ambitious goals of the International Rare Diseases Research Consortium (IRDiRC) by 2020 and beyond, RDRs must be designed with the agility to evolve and efficiently interoperate in an ever changing rare disease landscape, as well as to cater for rapid changes in Information Communication Technologies. In this paper, we contend that RDR requirements will also evolve in response to a number of factors such as changing disease definitions and diagnostic criteria, the requirement to integrate patient/disease information from advances in either biotechnology and/or phenotypying approaches, as well as the need to adapt dynamically to security and privacy concerns. We dispel a number of myths in RDR development, outline key criteria for robust and sustainable RDR implementation and introduce the concept of a RDR Checklist to guide future RDR development.

## Background

It is currently stated that there are over 7,000 rare diseases identified and reported which affect approximately 6-8% of the global population, although sound data is lacking. As such it is a public health issue, which requires an organised and systematic public health response, including accurate data for surveillance and monitoring, as well as for individual care. To obtain more reliable rare disease prevalence statistics in each country and to enable appropriate therapeutic translational research, Rare Disease Registries (RDR) are central [1-3]. International patient RDR are also critical to the pharmaceutical industry and there is now a very strong sense of urgency for national and regional–based registries to become coordinated in order to feed into these international registries, which often underpin clinical trials [4]. Furthermore, registries will provide information on the natural history of specific disorders and provide gene variation and disease phenotype data that will become increasingly important in evaluating new therapies and in determining patient access to what might be expensive treatments that often have strict access criteria though Government subsidy schemes. Unfortunately, to date, there are relatively few established national disease registries [3,5,6]. Recently, the groups of EURORDIS-NORD-CORD issued a Joint Declaration of 10 Key Principles for Rare Disease Patient Registries [7] and the European Union Committee of Experts on Rare Diseases published recommendations [8]. These principles are an invaluable guide for the creation of rare disease patient registries as well as to shape policy. They complement the main user’s guide in the field of registries for evaluating patient outcome [9]. A natural extension is to determine the metrics that could be used to measure the successful adoption of some of these principles.

A review of rare disease literature raises some important questions about RDR. First, there are semantic issues. For instance, what, if any, is the difference between a patient registry compared to a disease registry? How does this relate to a clinical registry? Is there any difference between a disease repository, disease registry, contact registry and a disease or patient database? What is the difference between a research 'cohort’ (eg EuroCYST [10]) and an audit registry (eg the UK Renal Registry [11])? What about their relatedness to national and ethnic mutation databases (NEMBDs) or locus-specific databases [12-14]? Second, what defines successful and sustainable interoperability between registries? Third, does the fact that a RDR system is available and permitted for download satisfy the term open source software or should other criteria be considered? Fourth, does the choice of software environment in which registries are implemented affect the ability of a RDR to be customised, extended or modified for evolving requirements? Fifth, what levels of security are employed, are they appropriate and is it possible to modify permissions and access privileges dynamically according to changing stakeholder needs? There are clearly some pre-conceived notions of disease registry development and in this paper we dispel three of these myths. In doing so, we highlight what we believe are important criteria that should be taken into consideration when developing RDR. We introduce the concept of a RDR Checklist to guide software development and project management best practices, which will allow rare disease stakeholders to better accommodate critical design issues that impact decision making.

### Dispelling rare disease registry development myths

#### Myth 1: technology is not a stumbling block

A commonly propagated message is that technical challenges are insignificant hurdles in the development of RDRs [1,5] and some Information Technology experts assure the rare disease community that technology is not the stumbling block [3]. We contend that technology choices, software architecture design and software development practices, to name a few, have a dramatic impact on issues such as software sustainability, legacy software support, ease of software modification/enhancements and interoperability. To emphasise the magnitude of the stumbling block facing software development in general, a recent European Union study considered one in eight information technology projects truly successful with the cost of project failure estimated to be 142 billion€ in 2004 [15]. This report lists a number of technical reasons for this failure including: inappropriate architecture; insufficient reuse of existing technical objects; inappropriate testing tools; inappropriate coding language; inappropriate technical methodologies; lack of formal technical standards; lack of technical innovation (obsolescence); misstatement of technical risk; poor interface specifications; poor quality code; poor systems testing; poor data migration; poor systems integration; poor configuration management; poor change management procedures; and poor technical judgment. We contend that many of these technical factors can manifest themselves in vendor lock-in [16]. In summary, in RDR development as in all software development, it is important to recognise that technology can, and often is, a stumbling block.

#### Myth 2: professional software developers are not required to develop Disease Registries

There is a significant difference between developing RDR that are grounded in professional software development processes versus under-resourced pilot projects that are undertaken to meet discrete internal user requirements with little, if any, engagement with external requirements/stakeholders. Professional software development is a complex undertaking that includes: i) appropriate software project management; ii) team-based software development; iii) well-structured, commented code; iv) version control; v) issue tracking; vi) documentation; and vii) software deployment instructions. In order to ensure value is delivered to the client, skilled software developers need to collaborate with end-users to produce working software which is technically excellent and builds in flexibility for modification should needs change [17].

Stakeholders undertaking RDR development should consider these issues so they can deliver viable software solutions while mitigating technical risk. In addition, it is instructive to examine a number of important considerations, such as whether the RDR should be a desktop application or an Internet-based application; developed on an open source or proprietary software platform; the use of a relational database management system or an alternative (eg. unstructured) data storage system; and the decision to deploy in a cloud environment or on physical ICT infrastructure. Other considerations are to ensure systems are capable of extensibility, interoperability, and security that are supported in a sustainable way. Once these decisions are made, a critical question becomes whether the chosen professional software development team possess the requisite skills and experience to adequately support the decisions made. The software development process requires expertise and it is costly and time consuming. It is interesting to note that in a self reported survey, undertaken by TREAT-NMD (http://www.treat-nmd.eu), the costs associated with developing national Spinal Muscular Atrophy registries in more than 30 countries, using a defined set of common data elements, were widely variable with some registries being established with ≤3000€, while others had funds in excess of 250,000€. The median amount of money invested to set up a registry was 20,000€ (Blanden, personal communication 2013).

#### Myth 3: open source is easy

Making software available for download is a relatively straightforward process. However, the simple ability to download software should not be confused with the complete and more complex process of open sourcing software. There are many instances of open source software that are difficult to install, come without detailed download instructions, release notes, version control, or documentation, and either do not work or fail with no available ongoing support. The quality of open source software relates to process: appropriate levels of documentation, strategies to capture community feedback, open and transparent installation processes, and the deployment process detailed. It is important to recognise that when a software team decides to open source software, they are not only making available their software to the broader community, they are also externalising their internal software development processes. This can be a paradigm shift in a software team’s operations as processes that come under scrutiny include deployment, testing, issue tracking, and accepting patches from the community. Open sourcing software is not as simple as uploading source code onto the Internet.

### System overview

A new approach to the design of disease registries to ensure access, security, privacy and the need for clinical sites across a given country has been developed. The Rare Disease Registry Framework (RDRF) enables access and registry of patients with clinical and genetic data often arising from different geographical locations. The approach adopted is readily applicable to other rare diseases [18].

#### Modular rare disease registry framework

The RDRF has been designed and implemented so that common features can be shared between registries. These common features include common data elements within what is referred to as *base modules*. Base modules might include: Patient Details, Medical History, Diagnosis Information, Genetic Variation, Working Groups and have agreed Common Data Elements (CDEs) providing conformity/interoperability of the data fields across platforms.

Patient Details and Medical History have been devised in consultation with patient advocacy groups. The CDEs conform to international standards such as TREAT-NMD. Base modules can be extended upon and customised for individual registries. For example, diagnostic information is tailored to each registry, since the required information varies significantly. New modules that are required by a specific registry can be contributed back to the base module set for use by other registries as required. As new registries are built, with each iteration and improvement, modules are able to be seamlessly incorporated within existing registries. As an example, a questionnaire module was created for the Australian Myotonic Dystrophy Registry (AMDR) which allows patients to directly enter information. The information entered is held in a 'quarantined’ region prior to being validated by a clinician. Once validated, this information is then incorporated into the registry. As this module was created for AMDR, it can now be loaded back into the other registries. This module can also be customized for web based patient registration and completion of self reported symptoms, which can be accepted or modified by a clinician at the next patient appointment.

In a similar way other modules created such as 2 factor authentication secure log-on, web-enabled consent and phenotyping approaches can be 'plugged-in’ as required. During the development of the RDRF, via professional agile software development processes, a refactoring process has now created a number of common modules that are shared between individual registries. Because of this flexible modular design and thanks to a collaboration with the Universal Mutation Database team, we will now be able to add specific genetic modules such as: predictions of the pathogenicity of reported exonic [19] or intronic [20,21] variations; genotype-phenotype correlations [22]; or even methods to facilitate new genotype based therapeutic approaches such as exon-skipping [23,24].

The RDRF graphical user interface is also modular so it can be easily customised for a given RD. A specific example is in the neuromuscular domain where, even as the national NMD registry grew, patient advocates from Spinal Muscular Atrophy (SMA) wanted a different user interface from Duchenne Muscular Dystrophy (DMD) or Myotonic Dystrophy. For SMA, the interface was modified to reflect real-time practice, i.e. it was aligned with how motor function is clinically captured by patients and their clinicians. These nuances were able to be accommodated without the need to modify the underlying architecture of the RDRF. Both national and international RD registries have now been built using this framework and they all have been informed by fundamental stakeholders such as patients and clinicians.

The RDRF has been developed to be able to automatically de-identify data when exported. The Australian DMD registry feeds into the TREAT-NMD international registry and additionally, where appropriate, we have designed interoperability to connect the Myotonic Dystrophy registry to both the TREAT-NMD core data and the Rochester Registries with equal degrees of interoperability for data exchange.

#### (i) Security and multi-level access is a key feature in the RDRF

The registry framework has two levels of access control, allowing fine-grained control of access: Groups (user-level) define the permissions granted to each user (functionality); and (ii) Working Groups restrict the content to which Group members have access. In addition, apart from Groups and Working Groups, permissions can also be set on an individual user basis. Working Groups might represent a clinic, hospital, a region or a state. They are private, and data is not shared between working groups. User groups such as treating Clinicians or Geneticists are added to a particular Working Group to allow them to work together. Within the RDRF, the security model consists of several layers: SSL based encryption of all traffic; password access to accounts; and logging of successful and failed user logins. In addition, the RDRF can also be configured to provide in-built IP address whitelisting and blacklisting, and two factor authentication. With these various levels of security, a significant level of confidence can be provided to end-users.

#### Interoperability

A key dimension to consider is the effort required for RDR to be interoperable. Tedious manual and repetitive data transfer between systems is not scalable. Fortunately, we can leverage other significant efforts to introduce the concept of Degrees of Interoperability into RDR development discussions. Specifically, NATO have developed four levels of interoperability that would be appropriate for rare disease research [25].

•Degree 1: Unstructured Data Exchange. Involves the exchange of human-interpretable unstructured data such as the free text found in operational estimates, analysis and papers.

•Degree 2: Structured Data Exchange. Involves the exchange of human-interpretable structured data intended for manual and/or automated handling, but requires manual compilation, receipt and/or message dispatch.

•Degree 3: Seamless Sharing of Data. Involves the automated sharing of data amongst systems based on a common exchange model.

•Degree 4: Seamless Sharing of Information. An extension of Degree 3 to the universal interpretation of information through data processing based on co-operating applications.

Understanding Degrees of Interoperability will enable decision makers, funders and research scientists to become aware of the effort required to sustain interoperability between RDR. For instance, if international registries require manual entry of unstructured patient/disease data (Degree 1) to interoperate with national registries, ultimately a decision needs to be made as to the financial viability to support this approach in the longer term, not to mention the known high risk of human error in this form of data exchange. If it is widely accepted, as it is in other fields, that Degrees 3 and 4 are the future directions for RDR harmonisation, strategic decisions can start to be made on interoperability not just between disease registries but also with other systems relevant in translational rare disease research, such as biobanks and integrative -*omics* analysis [26].

The RDRF has been recently customised for the group of Demyelinating nerve diseases (that includes both rare and common forms of Multiple Sclerosis) and includes facilities to upload MRI images and other additional information necessary for this group of diseases. The modular structure of the RDRF enables any RD registry deployed to evolve over time and maintain consistency with other registries. Because it is modular, it is relatively easily to customise the user interface without any significant software development effort.

#### Extensibility

**Figure 1:**
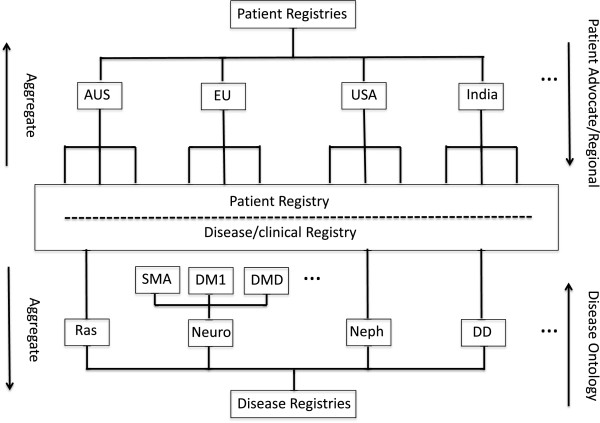
**Registry aggregation.** A schematic of how disease/patient registries might be aggregated.

A number of enhancements of the RDRF are underway. First, longitudinal phenotypes are being captured by a time-stamping functionality that captures a static record of the specific patient record at a given time before fields within the patient record are modified. Second, the RDRF is being refactored to enable aggregation of existing disease registries and enable the RDRF to be used for varied functionality as required, such as a patient registry, common registry, a disease-specific registry or even a clinical registry. A schematic of this approach is shown in Figure 1, which attempts to capture two broad existing registry domains, namely patient registries (country-centric) and disease/clinical registries (disease-centric). There is a need to aggregate disease registries and some complementary exemplars for this aggregation, based on disease ontologies, are suggested in Figure 1 and include: RASopathies; Demyelinating Diseases; Neuromuscular; Paediatric Nephrological disease; familial cancers; and paediatric cancers. Similarly, patient registries need to be aggregated from regional through to national and international levels. The centre box of Figure 1 attempts to capture the concept that registry frameworks might serve multiple purposes.

#### Disease registry requirements change over time

Registry system requirements evolve over time. For instance, a patient advocacy group might want to develop an initial general patient or contact registry for all diseases, which may need to morph into a registry for specific needs. However, if the software cannot support this, then a separate registry/registries will need to be established. Similarly, a given disease registry for a neuromuscular disorder might not be designed with other organ-specific clinical fields. If a neuromuscular patient on the RDR is diagnosed with a different rare disease (e.g. a haematological condition), should this additional clinical information be included in the existing neuromuscular disease registry, entered in a different registry, or both? Unfortunately, there is no systematic process to guide these decisions within the international rare disease community. The same remark also applies at the genetic level, which is frequently believed to be the “easy” part of the data. However, recent technological advances are leading to an evolution from single pathogenic variations associated with a patient to an expanded set of exome/genome wide genetic variation.

It is not difficult to anticipate that access restrictions will change over time. For any given user of the system, be they a Clinician, Geneticist, Patient Advocate, Curator, or Allied health worker, decisions will constantly be revisited on who gets access and on what grounds, what can be accessed and how, and where and when access can be gained. Through modular development, new features can be added and common modules created, but there is an ongoing need to refactor the code [17]. The modularity of the RDRF we have developed caters for ease in modifying access privileges and adding/customising modules.

#### RDRF checklist

In accordance with the above, we propose a new Checklist for RDR development. These key criteria for consideration in future RDR development are outlined in Table 1.

**Table 1:** RDR Checklist

***1****.* ***Technology choices***	***4****.* ***System design***
• Web-based or desktop application	• Customisable for
• Relational Database or unstructured data	○ a specific disease(s)
• Programming Language	○ patient registry
• Cloud deployment vs Physical ICT infrastructure	○ clinical registry
• Open source vs Proprietary	• Modular design
	○ new features
	○ new data elemets
	○ new ontologies
***2****.* ***Professional Software Development***	***5****.* ***Security***
• Appropriate software project management	• De-identification process
• Team-based software development	• Two factor authentication
• Well-structured, commented code	• Multi-level user access
• Version control	• Work groups
• Issue tracking	• Encryption
• Documentation	***6***. ***Sustainability***
• Software deployment instructions	• Ease of exchange
• Functional and Unit Testing	• Effort required
• Team-based development	• Future proofing
***3***. ***Interoperable***	***7***. ***Open source***
• Export/import functionality	• Appropriate levels of documentation
• Webservice API	• Strategies to capture community feedback
• Data standards	• Open and transparent installation processes
• Ontology	• Deployment process detailed
○ Data elements	
○ Disease elements	

Rare disease registry development RDR Checklist.

## Conclusions

The development of robust sustainable RDR is central to achieving the goals set by the International Rare Diseases Research Consortium (IRDiRC), which aim to have a diagnostic test for most rare diseases and 200 new therapies by 2020. Contemporary thinking is that a disease registry is only as good as the quality of the patient and disease information contained within it. In this paper, we contend that in the longer term, the quality of the system in which the data is contained becomes a significant bottleneck. There are a plethora of registries that differ in naming convention and functionality. Traditionally, not all existing registries are developed with interoperability and security in mind. Currently, there are significant overheads to validate and subsequently synchronise patient data from various regional, state-based, national registries into international resources as successfully demonstrated by the TREAT-NMD network of excellence that allows data collection from more than 40 countries. Patients provide informed consent, but unfortunately, there is often times incongruence between patient information and what clinicians and researchers require to assist in diagnosis and treatment; or the information may be siloed and inaccessible to appropriate allied health workers. This is not a viable situation going forward.

Fortunately, from our experience it is possible to design robust and sustainable RDR and to cater for the capture of vital information as our understanding of disease processes dramatically improves, through major advances in biotechnology and phenotyping. The captured data can not only drive research and development, but also improvements in clinical care, policy and population-wide outcomes for all people with rare diseases.

## Availability and requirements

 Project name: Rare Disease Registry Framework

Project home page: https://bitbucket.org/ccgmurdoch/disease_registry

Operating system(**s**): Linux

Programming language: Python

Other requirements: PostgreSQL, Apache (mod_wsgi)

License: GNU GPL v3

Any restrictions to use by non-academics: No

## Competing interests

The authors declare that they have no competing interests.

## Authors’ contributions

Original concept: MB and AH; Wrote manuscript: MB, AH, CB, KP, TH, SA, GB, TW, HD. All authors read and approved the final manuscript.

## Authors’ information

Matthew Bellgard: Professor Matthew Bellgard (BSc Hons, PhD in Computer Science) is Murdoch University’s Bioinformatics Chair and the Director of the Western Australian State Government Centre of Excellence, the Centre for Comparative Genomics (CCG). His scientific work has resulted in developments in the areas of pairwise sequence alignment and artificial intelligence, early detection of base composition differences in closely related bacterial species, whole genome sequence analysis and advances in the development of web-based integrated systems utilising high performance computing.

Christophe Beroud: Professor Christophe Béroud (PharmD, PhD) is leading the “Bioinformatics and Genetics” research team at INSERM UMR_S910. He has a strong experience in the fields of Locus Specific DataBases and patient registries. His work has also resulted in the creation of various systems dedicated to the human genome analysis including prediction of the pathogenicity of mutations and whole genome sequence analysis. He is also involved in rare diseases diagnosis and research in the fields of congenital muscular dystrophies and dystonia.

Kay Parkinson: Kay Parkinson is a qualified lawyer, she was the mother of two children with the ultra rare disease Alstrom Syndrome (AS). After their diagnosis when aged 15&18 respectively she founded ASUK charity. In 2006 the multi-disciplinary clinics she initiated were commissioned by the NHS, National Specialised Commissioning Group for Highly Specialised Services. ASUK were voted EURORDIS patient organisation of the Year 2013 for outstanding services to AS patients. She has now founded Alstrom Europe.

Tess Harris: CEO of the Polycystic Kidney Disease (PKD) Charity UK, President of PKD International and Secretary of the Ciliopathy Alliance. Tess is affected by ADPKD (autosomal dominant polycystic kidney disease) and represents PKD patients at UK and EU level. She chairs the UK Renal Registry Study Group for ADPKD and has experienced the challenges of establishing a patient/disease registry. She was formerly a businesswoman specialising in e-marketing and data protection.

Segolene Ayme: Ségolène Aymé is a medical geneticist, Emeritus Director of Research at the French Institute of Health and Medical Research (INSERM). She was the founder of Orphanet in 1997 (http://www.orpha.net), the webportal dedicated to rare diseases and orphan drugs. She chairs the European Union Committee of Experts on Rare Diseases (http://www.eucerd.eu) and the WHO Topic Advisory Group for Rare Diseases. She serves as Editor-in-Chief of the Orphanet Journal of Rare Diseases (http://www.ojrd.com). She is the project leader of “Support IRDiRC”, which provides the services of a scientific secretariat to the International Rare Diseases Research Consortium (http://www.irdirc.org).

Gareth Baynam: Associate Professor Gareth Baynam (MBBS, DCH, FRACP, PhD) is a Clinical Geneticist with an interest in rare disease registries including their coal-face clinical integration and their harmonisation with diagnostics and objective and scalable phenotyping techniques. He is a member of the Australian Rare Diseases Coordinating Committee, the Orphanet Australia National Advisory Board, Rare Voices Australia and President of the Western Australian Branch of the Human Genetics Society of Australia.

Tarun Weeramanthri: Professor Tarun Weeramanthri is Executive Director, Public Health and Clinical Services Division, in the Department of Health in Western Australia. He is an advocate for a national approach to rare diseases in Australia, and promotes the use of new technologies, including genomics, for broad public health goals.

Hugh Dawkins: Professor Hugh Dawkins (Director, Office of Population Health Genomics (OPHG), Public Health and Clinical Services Division, Department of Health Western Australia) is leading in the development of policies and plans to minimise the impact of genetic and rare diseases. He provides advice on genetic testing, screening, genetic privacy, stem cell therapy and the translation of new knowledge and technologies to improve in health services. OPHG is currently responsible for developing a state strategy for rare disease and preparing a scoping paper, at the request of the Australian Health Ministers’ Advisory Council on the need for a National rare disease plan in Australia.

Adam Hunter: Adam Hunter completed a BSc Honours in Computer Science at Murdoch University. Adam has over 10 years experience in ICT including software development in C and Java. He leads the CCG software development and infrastructure team. Current areas of focus include continuous integration, agile programming and high performance computing.
