# Correction: Dispelling myths about rare disease registry system development

**DOI:** 10.1186/1751-0473-9-4

**Published:** 2014-01-31

**Authors:** Matthew Bellgard, Christophe Beroud, Kay Parkinson, Tess Harris, Segolene Ayme, Gareth Baynam, Tarun Weeramanthri, Hugh Dawkins, Adam Hunter

**Affiliations:** 1 Centre for Comparative Genomics, Murdoch University, Perth 6150, Western Australia; 2 Faculté de Médecine de la Timone, INSERM UMR 910, Aix-Marseille Université, Marseille, France; 3 AP-HM, Département de Génétique Médicale, Hôpital d’enfants Timone, Marseille, France; 4 Alstrom Syndrome, 49 Southfield Ave, Paignton S, Devon TQ3 1LH, UK; 5 Polycystic Kidney Disease Charity (UK), PKD International, Ciliopathy Alliance, London, UK; 6 INSERM US14, ORPHANET 96 rue Didot, Paris 75014, France; 7 Genetic Services of Western Australia, King Edward Memorial Hospital, Perth, Western Australia; 8 School of Paediatrics and Child Health, University of Western Australia, Perth, Western Australia; 9 Institute for Immunology and Infectious Diseases, Murdoch University, Perth, Western Australia; 10 Office of Population Health Genomics, Public Health and Clinical Services Division, Department of Health, Government of Western Australia, Perth, Western Australia; 11 School of Pathology & Laboratory Medicine, University of Western Australia, Perth, Western Australia; 12 Curtin Health Innovation Research Institute, Curtin University of Technology, Perth, Western Australia

## Correction

After publication of this work [1], we noted that we inadvertently failed to include important Acknowledgments in our final version of the manuscript. Please see below the modification:

## Acknowledgements

The authors received funding from the Australian National Health and Medical Research Council (APP1055319) and EU FP7 Project (HEALTH.2012.2.1.1-1-C): RD Connect: An integrated platform connecting databases, registries, biobanks and clinical bioinformatics for rare disease research. The authors wish to acknowledge their involvement in the International Rare Disease Research Consortium.
